# Early screening of childhood ASD at primary care hospitals in western China: a multi-center study in Chengdu, Sichuan Province

**DOI:** 10.3389/fpubh.2026.1758181

**Published:** 2026-02-13

**Authors:** Wenxu Yang, Ying Zhang, Ping Yang, Chunxia Zhao, Chunhua Du, Junni He, Yanmei Cao, Jia Shang, Li Li, Yan Liu, Shenglan Wu, Xia Li, Xiujin Chen, Hai Lan, Hua Li, Xiang Kong, Hengli Li, Defang Mi, Jie Zhao, Yang Nie, JinXiu Gao, Tianyi Ma, Sophia Zuoqiu

**Affiliations:** 1 Chengdu Women’s and Children’s Central Hospital, School of Medicine, University of Electronic Science and Technology of China, Chengdu, China; 2 Jinniu Maternity and Child Health Hospital of Chengdu, Chengdu, China; 3 Chengdu Xindu Maternal and Child Health Care Hostpital, Chengdu, China; 4 Tianfu New District Huayang Community Health Service Center, Chengdu, China; 5 Longquanyi District of Chengdu Maternity and Child Health Care Hospital, Chengdu, China; 6 Wuhou District People’s Hospital of Chengdu, Wuhou District Health Hospital For Women and Children of Chengdu, Chengdu, China; 7 Dayi Maternal and Child Health Care Hospital, Chengdu, China; 8 Chengdu Chenghua District Health Care Hospital, Chengdu, China; 9 Wenjiang Maternal and Child Health Hospital, Chengdu, China; 10 Jintang County Maternal and Child Health Hospital, Chengdu, China; 11 Jinjiang Maternity and Child Health Hospital, Chengdu, China; 12 Chengdu Shuangliu District Maternity and Child Health Care Hospital, Chengdu, China; 13 Jianyang Maternal and Child Health Care Hospital, Chengdu, China; 14 Pengzhou Maternal and Child Health Care Hospital, Chengdu, China; 15 Qingbaijiang Maternal and Child Health Hospital, Chengdu, China; 16 Qionglai Maternal and Child Health Care Hospital, Chengdu, China; 17 Pujiang County Maternal and Child Health Hospital, Chengdu, China; 18 Dujiangyan Maternal and Child Care Service Hospital, Chengdu, China; 19 Chengdu Maternal and Child Health Hospital of Pidu District, Chengdu, China; 20 Chongzhou Maternal and Child Health Care Hospital, Chengdu, China; 21 Maternal and Child Health and Family Planning Service Center of Xinjin District, Chengdu, China; 22 Department of Industrial Engineering, Sichuan University-Pittsburgh Institute, Chengdu, China

**Keywords:** autism spectral disorder (ASD), child health care, prevalence, primary care, screening tools

## Abstract

**Objective:**

Autism spectrum disorder (ASD) prevalence is increasing rapidly, the screening efficacy of ASD at primary care hospital directly affects diagnostic efficacy. Currently, early ASD screening capacity in western China are limited. In this study we assessed the efficacies of four different ASD screening methods at primary care hospital in Chengdu.

**Methods:**

We recruited children 18–48 months through 20 primary care hospitals in Chengdu. Warning sign checklist [including autism warning sign (AWS) and social behavior observation (SB)], the Chinese-validated version of the Checklist for Autism in Toddlers (CHAT23), and autism behavior checklist (ABC) were utilized to identify potential ASD cases. Confirmation diagnosis was conducted at tertiary hospitals. Multivariate linear regression were conducted to compare efficacies of screening methods and to identify factors for positive ASD diagnosis.

**Results:**

In this real-world study, a total of 14,298 children attended the early ASD screening from 20 primary care hospitals in Chengdu. 13,458 (94.1%) children provided sufficient information and 3,502 (26.0%) children were concurrently screened with four ASD screening tools. The screen-positive rates varied across tools, in descending order, was AWS (3.1%), SB (2.7%), CHAT-23 (2.18%), and the ABC (1%). The overall referral rate reached 66.88%. Among the screened population who completed diagnostic follow-up, the detected prevalence of ASD was 0.79%. Regarding screening accuracy, the positive predictive values (PPV) were ABC (94.57%), SB (91.95%), and AWS (87.06%), CHAT-23 (84.72%) from highest to lowest. The multivariable logistic regression analysis showed factors associated with a final ASD diagnosis, adjusted for age, gender, and primary hospital. The ABC demonstrated the strongest independent association with ASD diagnosis (*β* = 8.40, SE = 1.92, *p* < 0.01). Age was also a strong significant predictor (*β* = 6.12, SE = 2.00, *p* < 0.01). Both the AWS (*β* = 3.43, SE = 1.85, *p* < 0.05) and CHAT-23 screening result (*β* = 2.63, SE = 1.58, *p* < 0.05) were independently and positively associated with the diagnosis. SB (*β* = 2.39, SE = 1.79) and gender (*β* = 1.35, SE = 1.64) were not statistically significant in the adjusted model.

**Conclusion:**

In summary, the ABC score and child’s age emerged as the most robust indicators of ASD in primary care hospitals. The effects of AWS and CHAT-23 support their utility as components in a multi-tool screening cascade. These findings support the practical value of integrated, tiered screening strategies in real-world public health settings.

## Introduction

Autism Spectrum Disorder (ASD) is a neurodevelopmental disorder with onset during early childhood (1). Children with ASD experience difficulties in social interaction and demonstrate repetitive or stereotyped behaviors (1). In the US, ASD prevalence was as high as 1 in 44 (2). In China, the national prevalence of ASD for children 6 to 12 years old was 0.7% (from July 2014 to December 2016) (3), while reported ASD prevalence for children 6 to 10 year old was 1% (from 2013 ~ 2014) (4). ASD was the primary reason for language and intellectual disabilities based on a national multi-center sampling survey among disabled children in China (5). While the average diagnostic age of ASD is 4.5 years (2), early intervention before that age could greatly improve the prognosis of ASD by helping the children to obtain better cognitive ability, language skill, and social adaptability (6).

However, due to insufficient understanding of ASD among parents and medical staff in primary care hospitals, children with ASD often missed the best time for early intervention. Autism spectrum disorder severity is significantly linked to higher out-of-pocket expenses (7). However, multiple cost-effectiveness analyses have demonstrated that providing early Intensive behavioral intervention in early childhood generally leads to lifetime cost savings (8). Therefore, beginning in 2015, the National Health Commission of the People’s Republic of China (formerly the Ministry of Health) started systematic screening for disabilities, including ASD during early childhood. Recently, Li and colleagues explored an improved autism screening model based on the maternal and child healthcare system in China (9), suggesting that improvement on the existing autism screening model and regional adaptivity of such improvement are possible.

Chengdu, a major metropolis located in southwest China, is the capital city of Sichuan Province. The city has a permanent resident population of 20,937,757 (10) and is the fourth most populous city in China. The city has 1,241,411 children under the age of 6 (6% of city’s population) (10). The city-wide maternal and child health care network in Chengdu consists of primary medical institutions based at community level, secondary hospitals at district/county level, and specialized tertiary hospitals (11). Currently, the secondary hospitals (hereon referred to as primary care hospitals) at district/county level serve as the main medical institutions where childhood ASD screening for children 0–6 years old is conducted. Suspected cases are referred to higher level medical institutions for confirmation using the gold standard diagnostic tool (ADOS, or ADIR). However, these primary care hospitals have not been able to accurately identify early signs of ASD due to limited knowledge and the lack of experience with screening methods, resulting in a negative feedback loop in medical knowledge for ASD screening, and further widening the knowledge gap between primary care hospital and specialized tertiary hospitals.

The current study aims to evaluate the feasibility and preliminary screening performance of a comprehensive protocol integrating both international and locally developed tools for the early screening of autism spectrum disorder within the routine child health services of primary care hospitals in Chengdu. We screened children using four different screening methods in 20 primary care hospitals that are part of the city-wide maternal and childcare network. Primary screening data from this study could provide real-world evidence for early childhood ASD diagnosis and could further strengthen scientific evidence for improving screening methods at primary care hospitals.

## Methods

### Study population

Between October 1, 2020 and May 31, 2021, we conducted screening for childhood ASD on children who were 18 to 48 months old at 20 maternal and child healthcare hospitals in Chengdu, Sichuan Province. For a complete list of participating hospitals, please see Supplementary Table S1. These participating hospitals were selected to ensure coverage within the established maternal and child health care system across Chengdu and each partiticpating hospital recruited children based on convenience sampling. Population distribution by district was obtained from publicly available Seventh National Census data (10). All parents (or guardians) were asked to provide consent for the study. A total of 14,298 children were recruited to participate in the screening, with a response rate of 94.1% (or 13,458 children). This study was approved by the Ethics Committee of the Chengdu Women’s and Children’s Central Hospital, School of Medicine, University of Electronic Science and Technology of China (2020-98).

### Screening methods and procedure

Four screening methods were utilized at the primary care hospitals for identifying potential ASD cases.

#### Autism warning sign

Autism warning sign (AWS) is an adaptation of Chinese warning sign checklist (WSC), a 44-indicator list established by a Chinese expert group for monitoring psychological and behavioral development, for 0–6 year old age group (12). AWS includes indicators for language development and social interaction from WSC because doctors are most likely to notice language deficiency, social behavior and communication issues during regular child health care (Supplementary Table S2). A positive result for aforementioned key indicators at key each age points means a positive screening result. Prior large-scale, communit-based screening in Shanghai, China established the feasibility and validity in applying AWS to children between 18 and 24 months of age (9).

#### Social behavior observational assessment

Social behavior (SB) observational assessment is an adaptation of WSC for 12 months old. SB contains two indicators: imitation of conventional gestures (waving goodbye or claps) and name response. Failure in either behavior result in a positive screening result. The reason why we chose social behavior as observation assessment is because that ASD patients have many non-verbal performance appear at young age and persist over time, with difficulty in imitating common social gestures (such as goodbye or clap), response to their names, pointing with a finger, interest in others and joint attention (13–15). The SB assessment is not administered in its original, standardized form for 12-month-olds. Instead, we have adapted its core elements to serve as a brief, observational screening probe within our study context. Specifically, we focused on two developmentally persistent social-communicative indicators derived from the tool: imitation of conventional gestures (e.g., waving goodbye, clapping) and response to name (13–15). Prior application of SB in Shanghai, China provided the evidence for the validity and reliability of using this screening tool for children who are 18–24 months old (9).

#### Chinese-validated version of the checklist for autism in toddlers

The Chinese-validated version of the Checklist for Autism in Toddlers (CHAT23) is an ASD screening method for children 18 ~ 24 months old (16). Part A of CHAT23 is composed of 23 questions for caregivers, and positive result for 6 out of the 23 indicators or 2 out of the 7 core indicators results in a positive screening result. Part B is a short observation consists of 4 questions evaluated by doctors during face-to-face interviews with children. Two or more abnormal results result in a positive screening result. Failure in both part A and part B results in a positive screening result.

#### The autism behavior checklist

The Autism Behavior Checklist (ABC) is a questionnaire for caregivers of children 18–35 months old (17, 18). The Chinese version of ABC is a 5-part and 57 indicators questionnaire covering sensory behavior, social relating, body and object use, language and communication skills, and social and adaptive skills (19). A screening cutoff score of 53 is used to indicate a positive screening result, while a total score greater than or equal to 68 can support the diagnosis of autism (20).

#### Screening procedure

**Figure 1:**
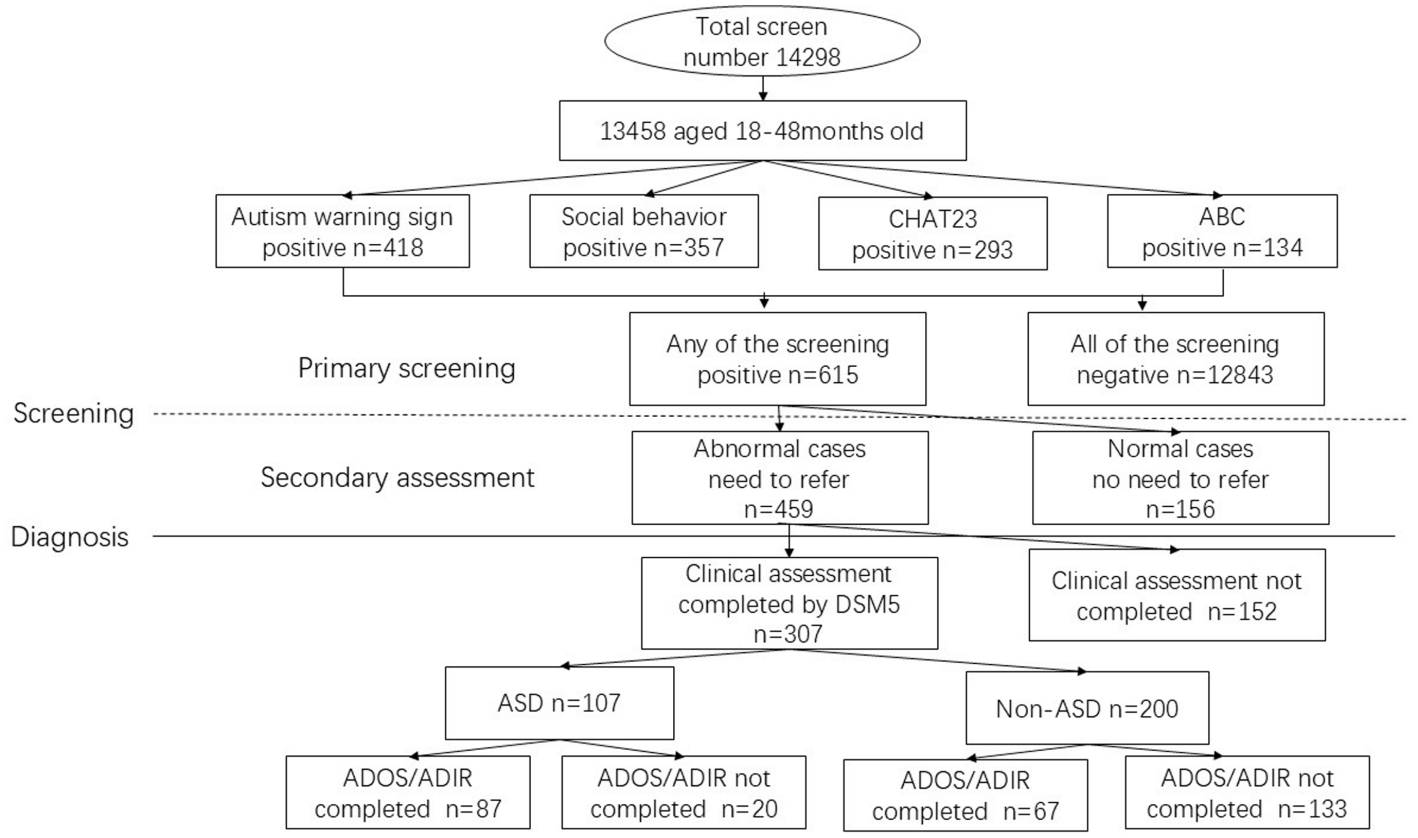
Flowchart of the two-stage ASD screening and diagnostic procedure in Chengdu.

This study utilized a two-stage process consisted of initial screening at primary care hospitals and confirmation diagnostic assessment at tertiary hospital (Figure 1). Primary screening for children 18–48 months utilized four screening methods (autism warning signs, social behavior observational assessment, CHAT23, and ABC). A positive result from any one of the four screening methods was defined as a positive case and led to a secondary assessment by a different doctor. An affirmative assessment result from a the secondary doctor leads to a referral to tertiary hospital. Children who screened positive were assessed face-to-face in a second evaluation conducted by a different physician (independent from the initial screening physician). This assessment primarily relied on clinical interviews and behavioral observations. If the secondary assessment result remained positive, the child was referred to a tertiary hospital. The specialist physicians conducted the final diagnosis based on clinical judgment by DSM-5 criteria, either in person or via telephone. The Gold-standard tools (ADOS, ADI-R) were used. The validity of telephone assessments is supported by prior research (9), which confirmed no statistically significant difference in ASD screening results between trained physicians conducting assessments via telephone and those conducted face-to-face.

### Diagnostic procedure

A positive screening result based on any aforementioned screening method triggers a referral to tertiary hospital for confirmational diagnosis. The diagnosis of Autism Spectrum Disorder (ASD) was established by a trained team of qualified clinicians in strict accordance with the diagnostic criteria of the Diagnostic and Statistical Manual of Mental Disorders, Fifth Edition (DSM-5) (21). To inform and support the clinical diagnostic process, comprehensive assessments were conducted using standardized instruments, including the Autism Diagnostic Observation Schedule (ADOS) (22) and the Autism Diagnostic Interview-Revised (ADI-R) (23). Consequently, the diagnostic assessment for each participant fell into one of the following three assessment support scenarios: (1) Diagnosis was based on clinical judgment using DSM-5 criteria without the use of the ADOS or ADI-R. (2) Diagnosis was informed by clinical assessment plus results from the ADOS. (3) Diagnosis was informed by clinical assessment plus results from both the ADOS and the ADI-R. Regardless of the assessment tools used, the final clinical diagnosis was a binary categorization: ASD or non-ASD, in accordance with DSM-5 criteria. Children with positive result from primary screening can choose voluntarily to be diagnosed in any of the tertiary hospitals located in Chengdu.

### Quality control

Prior to the study, we conducted training for physicians and staff from all 20 participating hospitals. They were informed of research procedure, standardized assessment procedure, data recording procedure and other relevant processes. During the study, we conducted monthly data assessment to ensure data quality. Additionally, we conducted monthly on-site check at the participating hospitals to monitor screening procedure and diagnostic assessment. During the study, we established a communication platform to receive questions from participating hospitals and all questions and issues were resolved promptly. At the end of 6th month of the study, we held the second follow-up training session and quality control.

### Statistical analysis

Data summary and data analysis were conducted in R language (24). Differences between groups were assessed by Chi-Square test and unpaired *t*-tests at statistical significance level of 0.05. The sensitivity (Se), specificity (Sp), and positive predictive value (PPV) were also calculated to assess diagnostic accuracy based on total positive (TP), total negative (TN), false positive (FP) and false negative (FN) for each of the four screening tools (Supplementary Table S3). Multivariate linear regression model was constructed in R language with package lme4 (25) to analyse potential factors associated with ASD diagnosis. Screening method was the primary dependent variable of interest. The model was adjusted for age, gender, and hospital. The outcome variable (ASD diagnosis) was tested for homoscedasticity across primary hospitals, and primary hostpital was included in the regression to control for potential clustering effect. Age was included as a control variable given its significant relationship with ASD outcome based on previous studies (26–28, 39).

## Results

### Study population characteristics and ASD prevalence

Out of recruited 14,298 children from 20 primary care hospitals in Chengdu, 13,458 (94.1%) provided sufficient information (Figure 1). The study population consisted of more males (7,078, or 52.6%) than females (6,380, or 47.4%), with an average age of 21.18 ± 3.46 months. A total of 107 children were diagnosed with ASD, including 83 (1.2%) males and 24 (0.4%) females, with an average age of 30.80 ± 7.55 months. The estimated ASD prevalence of this study was 0.79% in Chengdu. The prevalence in males was higher than that in females, with a male to female ratio of 3.5:1(*χ*^2^ = 74.54, df = 1, *p* < 0.001). Chi-square analysis revealed a significant difference in ASD diagnosis rates across hospitals (*χ*^2^ = 32.87, df = 19, *p* = 0.0100). The significant variation suggests differences in screening implementation, diagnostic practices, or population characteristics across hospitals warrant further investigation.

### ASD screening rates and diagnostic prevalence

A total of 615 (4.6%) children receive positive screening result. Specifically, 418 (3.1%), 357 (2.7%), 293 (2.18%) and 134 (1.0%) children received positive result by autism warning sign(AWS), social behavior observational assessment (SB), CHAT 23 and ABC, respectively (Table 1). Secondary assessment found 459(74.6%) children with possibility to develop autism and these potential positive cases were referred to tertiary hospitals.

**Table 1:** Summary of screening results, referral and final diagnoses of ASD.

Primary hospital	Screening *N*	Positive cases AWS *N* (%)	Positive cases SB *N* (%)	Positive cases CHAT23 *N*(%)	Positive cases ABC *N* (%)	Need to refer *N*	Acutal referred *N*	Total referral (%)	ASD diagnosis rate *N*(%)	95% CI (Wald)
Jinniu	1934	16(0.8%)	18 (0.9%)	21 (1.09%)	4 (0.2%)	26	19	73.08%	6 (0.3%)	(0.062, 0.058)
Xindu	1,414	158 (11.2%)	94 (6.6%)	47 (3.32%)	37 (2.6%)	107	47	43.93%	13 (0.9%)	(0.421, 1.417)
Longquanyi	922	24 (2.6%)	28 (3.0%)	54 (5.86%)	13 (1.4%)	41	30	73.17%	15 (1.6%)	(0.809, 2.445)
Jinjiang	880	22 (2.5%)	19 (2.2%)	19 (2.16%)	10 (1.1%)	26	25	96.15%	6 (0.7%)	(0.137, 1.226)
Tianfu New Area	855	13 (1.5%)	23 (2.7)	27 (3.16%)	10 (1.2%)	48	26	54.17%	9 (1.1%)	(0.368, 1.738)
Dayi	827	14 (1.7%)	9 (1.1%)	8 (0.97%)	4 (0.5%)	15	13	86.67%	7 (0.8%)	(0.221, 1.472)
Chenghua	824	11 (1.3%)	10 (1.2%)	6 (0.73%)	3 (0.4%)	11	10	90.91%	6 (0.7%)	(0.147, 1.310)
Jintang	721	3 (0.4%)	3 (0.4%)	4 (0.55%)	2 (0.3%)	5	3	60.0%	1 (0.1%)	(−0.133, 0.411)
Wuhou	690	17 (2.5%)	15 (2.2%)	13 (1.88%)	11 (1.6%)	18	13	72.22%	5 (0.7%)	(0.091, 1.359)
Shuangliu	643	21 (3.3%)	42 (6.5%)	11 (1.71)	3 (0.5%)	37	20	54.05%	8 (1.2%)	(0.386, 2.103)
Wenjiang	632	20 (2.7%)	23 (3.6%)	16 (2.53%)	13 (2.1%)	22	16	72.73%	9 (1.4%)	(0.499, 2.350)
Jianyang	515	28 (5.4%)	13 (2.5%)	22 (4.27%)	4 (0.8%)	22	20	90.91%	6 (1.2%)	(0.236, 2.094)
Pengzhou	504	12 (2.4%)	6 (1.2%)	6 (1.19%)	5 (1%)	16	13	81.25%	1 (0.2%)	(−0.191, 0.588)
Qingbaijiang	480	17 (3.5%)	15 (3.1%)	11 (2.29%)	3 (0.6%)	15	13	86.67%	5 (1.0%)	(0.131, 1.952)
Qionglai	394	4 (0.1%)	4 (1.0%)	0 (0%)	1 (0.3%)	4	4	100.0%	1 (0.3%)	(−0.245, 0.752)
Pujiang	330	9 (2.7%)	3 (0.9%)	2 (0.61%)	0 (0.0%)	6	5	83.33%	1 (0.3%)	(−0.292, 0.898)
Dujiangyan	310	6 (1.9%)	15 (4.8%)	9 (2.90%)	4 (1.3%)	14	8	57.14%	2 (0.6%)	(−0.250, 1.540)
Pidu	270	12 (4.4%)	8 (3.0%)	10 (3.7%)	2 (0.7%)	10	9	90.0%	2 (0.7%)	(−0.287,1.768)
Chongzhou	232	7 (3%)	6 (2.6%)	4 (1.72%)	3 (1.3%)	10	9	90.0%	2 (0.9%)	(−0.334, 2.058)
Xinjin	81	4 (4.9%)	3 (3.7%)	3 (3.7%)	2 (2.5%)	6	4	66.67%	2 (2.5%)	(−0.962, 5.901)
Total	13,458	418 (3.1%)	357 (2.7%)	293 (2.18%)	134 (1.0%)	459	307	66.88%	107 (0.79%)	(0.645, 0.945)

AWS, autism warning sign; SB, social behavior assessment. N is number of individuals.

A total of 307 (307/459, 66.88%) children referred completed clinical diagnosis at tertiary hospitals. Of those completed clinical diagnosis, 107 (107/307, 34.9%) were diagnosed with ASD (Figure 1), and 200 (200/307, 65.1%) were diagnosed as non-ASD (including high risk for ASD but did not meet diagnostic criteria, intellectual disability and developmental language disorder). Of the 107 confirmed ASD cases, 87(87/107, 81.3%) were evaluated by either ADOS or ADOS+ADI-R, and 20(20/107, 18.7%) were only made by clinical judgment using DSM-5 criteria. Of these non-ASD, 67(67/200, 33.5%) were evaluated by either ADOS or ADOS+ADI-R, and 133(133/200, 66.5%) were only made by clinical judgment using DSM-5 criteria. Children with eventual positive diagnosis (ASD cases) and children who received a negative diagnosis (non-ASD) obtained statistically different ADOS and ADIR scores (*p* < 0.001) (Table 2), except in the “play” domain (C domain, *p* = 0.256).

**Table 2:** Confirmation diagnosis using ADOS and ADI-R domain scores.

Standardized instruments	ASD Mean (SD)	Non-ASD Mean (SD)	*T*-test
ADOS			
A	6.3 (1.1)	5.1 (1.9)	4.82*
B	9.7 (1.8)	7.5 (2.2)	6.77*
A + B	16.0 (2.5)	12.6 (3.4)	6.93*
C	3.0 (1.0)	2.8 (1.0)	1.14
D	1.5 (0.7)	0.3 (0.6)	11.74*
ADI-R			
A1	16.5 (4.2)	12.1 (3.6)	5.35*
B1	11.5 (2.6)	8.9 (2.6)	4.80*
C1	2.7 (1.5)	1.6 (1.2)	4.02*

ADOS algorithm: A, communication; B, reciprocal social interaction; C, play; D, restricted, repetitive, and stereotyped behavior; A + B, communication+ reciprocal social interaction. ADI-R algorithm: A1, social interactions; B1, communication; C1, restricted, repetitive and stereotyped behaviors. *p < 0.001.

### Efficacy of four screening methods

Not all children completed all four tools, primarily due to practical constraints such as on-site feasibility, parental compliance, and the child’s condition. The diagnostic utility of screening methods for distinguishing ASD from non-ASD children was assessed on subjects with valid data in all four screening methods (total *n* = 3,502, ASD *n* = 62, non-ASD *n* = 3,440) (Table 3). These four screening tools were able to correctly classify ASD and non-ASD samples. ABC tool showed the highest specificity (99.77%) and positive predictive value (94.57%). ASD warning sign exhibited the highest sensitivity (56.06%).

**Table 3:** Summary of AUC, sensitivity (SE), specificity (SP) and positive predictive value (PPV) for discriminating ASD and non-ASD from current and previous studies.

Referred studies	Screening method	AUC	SE (%)	SP (%)	PPV (%)
Current study					
	AWS	0.775 (0.7714–0.7785)	56.06 (51.14–60.87)	98.94 (98.51–99.24)	87.06 (82.38–90.63)
	SB	0.771 (0.7674–0.7745)	54.80 (49.87–59.63)	99.39 (99.05–99.61)	91.95 (87.77–94.79)
	CHAT23	0.739 (0.7357–0.7429)	48.99 (44.10–53.90)	98.87 (98.44–99.19)	84.72 (79.49–88.80)
	ABC	0.653 (0.6496–0.6563)	30.81 (26.46–35.52)	99.77 (99.54–99.89)	94.57 (89.22–97.35)
Previous studies					
Huang et al. (12)	WSC	—	82.2	77.7	—
Qiu et al. (36)	CHAT23-A	0.934	92.0	90.0	—
Li et al. (9)	CHAT23	—	—	—	48.5
Wu et al. (37)	CHAT23	—	94.1	88.4	50.0
Wong et al. (16)	CHAT23	—	93.1	76.8	73.6
Ren et al. (38)	CHAT23	—	97.1	96.1	85.7
Qiu et al. (35)	CHAT23	—	92.6	96.3	—
Marteleto et al. (17)	ABC	0.900	57.9	94.7	—
Özdemir et al. (18)	ABC	—	86.6	81.7	—

AWS, autism warning sign; SB, social behavior; CHAT23, the Chinese-validated version of the Checklist for Autism in Toddlers; ABC, autism behavior checklist; WSC, warning signs checklist; Se, Sensitivity; Sp, Specificity; PPV, Positive Predictive Value; AUC, area under the curve. The PPV for ASD of screening is defined as the proportion of children screened as positive who received an ASD diagnosis, excluding the lost to follow up, and divided by the total number of screen-positive cases.

### Regression result

Multivariate model was constructed to understand the association between ASD diagnosis and screening methods while adjusting for age at screening, gender and primary hospital (Table 4). The regression result indicates that age was significantly associated with the probability of receiving a positive ASD diagnosis result, with each year associated with an increase of more than 6 times the likelihood of being diagnosed with ASD (*p* < 0.01). Screening results using Austism Spectrum Warning Sign, ABC and CHAT 23 tools were also significantly associated with eventual positive diagnosis of ASD. Social Behavior screen tool was not significantly associated with the eventual austism diagnosis.

**Table 4:** Summary of logistic regression.

Variables	Estimate (SE)
Autism spectrum warning sign	3.4281 (1.8500)*
ABC	8.3981 (1.9188)**
CHAT 23	2.6277 (1.5779)*
Social behavior	2.3900 (1.7851)
Age	6.1227 (1.9951)**
Gender	1.3458 (1.6413)

Model outcome is final austism spectrum diagnosis (positive or negative). Model adjusted for: age, gender, primary hospital. Significance level: “***” 0.001 “**” 0.01 “*” 0.05.

## Discussion

### The efficacies of screening methods

The four screening tools in this study, AWS, SB, CHAT-23 and ABC, varied in their efficiacies in detecting early signs of ASD (Table 3). Notably, the ABC scale demonstrated the highest specificity and PPV, whereas CHAT-23 showed the lowest corresponding values, reflecting their differing design focuses (9, 29–31): CHAT-23 emphasizes high sensitivity to reduce missed diagnoses in initial screening, while ABC focuses on comprehensive behavioral observation to improve specificity and PPV by better excluding non-cases. This suggests that selecting appropriate tools or combinations based on screening phase objectives in primary care practice, such as high sensitivity for initial screening versus high specificity for follow-up, can optimize overall screening efficiency.

We found that the likelihood of detecting ASD increases with age, which is consistent with previous research (26, 32–34). Common reasons include atypical symptoms in younger children, the absence of intellectual disability in some cases, and insufficient awareness among parents/primary care physicians. However, as age increases, the ability of screening tools to capture core symptoms improves, leading to a higher positive predictive value (9, 35).

### Referral rate and diagnosis rate

Our study’s referral rate was 66.88%, which was slightly higher than previously reported using the standard primary screening model, but lower than that of the improved screening model by Li and colleauges (9). Total referral rate did not directly translate into diagnosis rate for ASD in our study. For example, the total referral rates was 54.17% in Tianfu New Area and 43.93% in Xindu, with the higher diagnosis rates of ASD nearly more than 1% for this two region, but the total referral rate of Qionglai was 100%, with the lower diagnosis rates of 0.3% (Table 1). This study observed a phenomenon of “low referral rate but high diagnostic confirmation rate” for autism spectrum disorder (ASD) screening in primary care hospitals. The core reasons are that some primary institutions employ a combination of high-specificity screening tool and professional clinical judgment, achieving accurate initial screening and effectively identifying a high-risk population. For example, hospitals in Tianfu New Area and Xindu were proactive in multiple attempts in telephone follow-up with screening positive cases; such attempts increases parents’ awareness of the importance of referrals and consequently increased number of actual referral visits. Concurrently, barriers to accessing diagnostic resources (e.g., difficulty in making appointments, long distances) further select for families with the highest compliance and greatest concern, whose children often present with more typical symptoms, thereby increasing the final confirmation rate. From a public health perspective, this model demonstrates the potential advantage of the primary care “gatekeeper” role in improving resource-use efficiency and avoiding over-referral. However, it is also crucial to mention that overly stringent screening criteria may lead to delayed identification of atypical, mild, or early-stage ASD cases.

Furthermore, the lack of significant differences in the “PLAY” component of ADOS is an interesting result. The diagnostic value of the ADOS lies in the integration of multi-domain information. Our study showed that while there was no significant difference in the play section, significant differences were observed in other domains such as social interaction, communication, and restricted/repetitive behaviors (Table 2). This aligns with the core diagnostic criteria of ASD, where deficits in social communication are essential, while impairments in play ability are common but not absolute or specific manifestations. Furthermore, among the 107 children diagnosed with ASD, the mean age of the ASD group diagnosed using the ADOS based on DSM criteria (*n* = 87) was 2.66 years, compared to 2.16 years in the non-ASD group (*n* = 67) (Figure 1). The severity and ability spectrum of ASD are broad, and some children may retain certain basic play skills.

The detected prevalence of ASD in the screened population of this study among children 18–48 months old was estimated to be 0.79%, with a male to female ratio of 3.5:1. These findings are consistent with the earlier reports (3, 4), especially the study by Zhou and colleagues conducted in 2016 that included only two districts in Chengdu (Tianfu New Area and Pidu) using a similar multi-center study approach; their results indicated that ASD prevalence among school-age children 6–12 was 0.7% (3). However, because not all referred children received diagnostic assessment in our study (diagnostic assessment at tertiary hospital was voluntary), the estimated prevalence reported by our study could be lower than the true prevalence.

### Major factors for ASD diagnosis

In our study, we found that the ABC, age, AWS and CHAT-23 have significant positive relationships with ASD diagnosis. Our study deployed a two-stage approach with initial screening (stage 1) and confirmation assessment (stage 2). During the confirmation assessment stage, ASD diagnosis was conducted using gold standard tools ADOS and ADIR. Previous studies have shown that ADOS has strong stability and the ADIR has higher sensitivity as children age (27, 28). Additionally, the core symptoms could become more serious as children age and could be more obvious during diagnosis assessment (15). From a public health perspective, the descending strength of association (ABC > age > AWS > CHAT-23) with ASD diagnosis reveals a natural screening cascade. This evidence directly supports implementing a tiered screening strategy in primary care hospitals. For example, initial screening with high-sensitivity tools (e.g., CHAT-23/AWS), followed by secondary evaluation of screen-positive children using the age-calibrated ABC scale to prioritize those at highest risk for diagnostic referral. These findings provide the foundation for developing a localized, multivariable risk prediction model to further enhance screening precision at the community level.

### Strengths and limitations

This study represents the first large-scale, parallel comparison of multiple screening tools, including the AWS within a primary healthcare system. Secondly, it provides critical front-line data to inform the development of China’s “early screening and intervention system for autism.” The real-world data from this study provided more accurate evidence for the evaluation of ASD screening efficacy in primary care hospitals. The study demonstrates a design and analytical framework for conducting high-quality public health research under real-world, non-ideal conditions. However, our analysis was limited by several unobserved confounders, such as socio-economic status and education level of parents; we could not obtain such data for the current study. The lack of such information could confound our results towards null and potentially undermine the relationship between screening tools and outcome. Additionally, our study utilized convenience sampling approach and participating in our study was voluntary, therefore, our study population could be different than the general children population, thus our results, especially estimated prevalence, could have limited representativeness. The number of children recruited through different primary hospitals also varied and was unbalanced, which hinders the comparability of prevalence estimates across districts and potentially affects the generalizability of our results. Moreover, in this research, approximately one-third of the referred children were lost to follow-up prior to completing the diagnostic assessment. After a positive screening result, parents often refuse further diagnosis for various reasons. Common ones include long wait times and the child not being in an optimal state (especially among children aged 2–3 years) during the initial test. Although we attempted supplemental telephone follow-up for a subset of these cases, the data obtained were neither complete nor systematic enough for formal inclusion. Due to the lack of information the loss-to-follow-up information, we could not conduct further anlaysis to compare the population that completed the diagnostic process and the ones who did not. This limitation could result in inflation of PPV, and thus yielding a higher PPV value. Furthermore, while we adjusted for age as a continuous confounder in multivariate models, we were unable to provide age-stratified estimates of screening performance (e.g., sensitivity, specificity) due to the limited number of confirmed ASD cases within specific age strata. Future studies with larger samples are needed to precisely evaluate the performance of these tools within narrower age windows.”

## Conclusion

The current study focuses on exploring the prevalence of childhood ASD based on four screening tools (ABC score, AWS, SB and CHAT-23) applied through 20 primary maternal and child healthcare hospitals in Chengdu, China. The results show an estimated prevalence of 0.79% among 13,458 children between 18 months and 48 months of age. Statistical analysis indicate that ABC score, AWS, and CHAT-23 were significantly associated with ASD diagnosis, whie age also has a significant positive relationship with ASD diagnosis. Though the interpretation and generalizability are limited by sampling strategy and the lack of confounding factors (such as socio-economic status), findings from this study still provide useful insight as it is the first city-wide evaluation of ASD prevalence in Chengdu based on primary screening data. Findings from this study could provide scientific basis for implementating customized diagnostic protocols suitable for primary hospitals.

## Data Availability

The raw data supporting the conclusions of this article will be made available by the authors, without undue reservation.
