# Supplementary material for: Early screening of childhood ASD at primary care hospitals in western China: a multi-center study in Chengdu, Sichuan Province
Source: Front Public Health. 2026 Feb 13;14:1758181. doi: 10.3389/fpubh.2026.1758181 (PMC12945995; doi:10.3389/fpubh.2026.1758181)
Supplement: Supplementary file 1 [file Supplementary_file_1.docx]

Table S1. List of Participating Women and Child Care Hospitals

| **Participating Hospital** | **Corresponding District/County/City*** |
| --- | --- |
| Chenghua Women and Children Healthcare Center | Chenghua District |
| ChongzhouWomen and Children Healthcare Center | Chongzhou City |
| Dayi Women and Children Healthcare Center | Dayi County |
| Dujiangyan Women and Children Healthcare Center | Dujiangyan City |
| Jianyag Women and Children Healthcare Center | Jianyang City |
| Jinjiang Women and Children Healthcare Center | Jinjiang District |
| Jinniu Women and Children Healthcare Center | Jinniu Disctrict |
| Jintang Women and Children Healthcare Center | Jintang County |
| Longquangyi Women and Children Healthcare Center | Longquangyi District |
| Pengzhou Women and Children Healthcare Center | Pengzhou City |
| Pidu Women and Children Healthcare Center | Pidu District |
| Pujiang Women and Children Healthcare Center | Pujiang County |
| Qingbaijiang Women and Children Healthcare Center | Qingbaijiang District |
| Qionglai Women and Children Healthcare Center | Qionglai City |
| Shuangliu Women and Children Healthcare Center | Shuangliu District |
| Wenjiang Women and Children Healthcare Center | Wenjiang District |
| Wuhou Women and Children Healthcare Center | Wuhou District |
| Xindu Women and Children Healthcare Center | Xindu District |
| Xinjin Women and Children Healthcare Center | Xinjin District |
| Huayang Community HealthCare Center | Tianfu New Area** |

*City here refers to county-level administrative district that is part of metropolitan Chengdu. **Tianfu New Area is a special administrative district.

Table S2. Autism Warning Sign Indicators Used in Current Study

| **Age (Month)** | **Warning Signs** | **Indication** | **Explanation** |
| --- | --- | --- | --- |
| 18 | Can't consciously call dad or mam | L | Can’t consciously and correctly pronounce when seeing mom or dad or caregivers |
|  | Failure to point at people or object as required | SI | Can’t point to familiar people or objects at home as adults request |
|  | no eye contact with people | SI | No eye contact or avoiding eye contact when adults talk to them |
| 24 | Can't say the name of three items | L | Can't name 3 familiar objects, such as lamp, car, cup |
|  | unable to do simple instruction | SI | Can't do simple things as parents request such as “pick up things” |
| 30 | Can't say a phrase | L | Can't say phrases with verb-object or subject-verb, such as "get some water", "go out to play" |
|  | Restricted, repetitive, and stereotyped interest | SI | Play with one or two objects or repeating the same action in a fixed manner for a long time, such as playing with the wheels of the car |
|  | Unable to signal urination and defecation | SI | Can’t use gestures or words to ask parents for help when she/he has to urinate or defecate |
| 36 | Can't say her/his own name | L | Can't pronounce her/his name or nickname correctly |
|  | unable to play pretend games such as" riding a horse with a stick | SI | Can’t do pretend play |
| 48 | Can't say sentences with adjectives | L | Say sentences that do not contain adjectives such as “I have red balloons” |
|  | Unable to wait or take turns as required | SI | Can’t wait or take turns in order as request when playing or doing things, |

The warning signs indicative of abnormal language and social interaction function from the WSC [12] were selected to be AWS for children 18-48 months group in our study. L, language; SI, social interaction.

Table S3. Contingence Table for Calculating Specificity, Sensitivity, Area Under the Curve and Positive Predictive Value (Positive ASD diagnosis =62, non-ASD =3440).

| **Screening Tools** | **TP** | **TN** | **FP** | **FN** |
| --- | --- | --- | --- | --- |
| CHAT-23 | 194 | 3071 | 35 | 202 |
| ABC Score | 122 | 3099 | 7 | 274 |
| Social Behavior | 217 | 3087 | 19 | 179 |
| AWS* | 222 | 3073 | 33 | 174 |

AWS: ASD warning sign
